# Serological Response and Relationship with Gender-Sensitive Variables among Healthcare Workers after SARS-CoV-2 Vaccination

**DOI:** 10.3390/jpm12060994

**Published:** 2022-06-18

**Authors:** Roberto Cangemi, Manuela Di Franco, Antonio Angeloni, Alessandra Zicari, Vincenzo Cardinale, Marcella Visentini, Guido Antonelli, Anna Napoli, Emanuela Anastasi, Giulio Francesco Romiti, Fabrizio d’Alba, Domenico Alvaro, Antonella Polimeni, Stefania Basili, SAPIENZAVAX Collaborators

**Affiliations:** 1 Department of Translational and Precision Medicine, Sapienza University of Rome, 00185 Roma, Italy; roberto.cangemi@uniroma1.it (R.C.); marcella.visentini@uniroma1.it (M.V.); giuliofrancesco.romiti@uniroma1.it (G.F.R.); domenico.alvaro@uniroma1.it (D.A.); 2 Department of Internal Clinical, Anesthesiologic and Cardiovascular Sciences, Sapienza University of Rome, 00185 Roma, Italy; manuela.difranco@uniroma1.it; 3 Department of Experimental Medicine, Sapienza University of Rome, 00185 Roma, Italy; antonio.angeloni@uniroma1.it (A.A.); alessandra.zicari@uniroma1.it (A.Z.); emanuela.anastasi@uniroma1.it (E.A.); 4 Department of Medico-Surgical Sciences and Biotechnologies, Sapienza University of Rome, 00185 Roma, Italy; vincenzo.cardinale@uniroma1.it; 5 Department of Molecular Medicine, Sapienza University of Rome, 00185 Roma, Italy; guido.antonelli@uniroma1.it (G.A.); anna.napoli@uniroma1.it (A.N.); 6 General Direction, Umberto I Hospital, 00161 Roma, Italy; f.dAlba@policlinicoumberto1.it; 7 Department of Oral and Maxillofacial Science, Sapienza University of Rome, 00185 Roma, Italy; antonella.polimeni@uniroma1.it or

**Keywords:** COVID-19, mRNA vaccine, BNT162b2, SARS-CoV-2, antibody response

## Abstract

Vaccine-induced immunity is a key strategy in the long-term control of the COVID-19 pandemic. The aim of our study was to explore the relationship between mRNA vaccine-induced antibodies and gender-sensitive variables among healthcare workers. Two thousand-sixty-five volunteers who received the BNT162b2 vaccine were enrolled in the study and followed up. Demographic, clinical, and social variables (educational level, marital status, occupation, childcare) were evaluated through a self-administered questionnaire. Anti-Spike (S) IgG were measured at 1 month (T1) and at 5 months (T2) after the second vaccine dose. At T1, median anti-S IgG values were 693 [394–>800] AU/mL (1 AU = 2.6 BAU). Values > 800 AU/mL (2080 BAU/mL) were directly associated with a previous COVID-19 (*p* < 0.001) infection and inversely with age (*p* < 0.001), smoking habit (*p* < 0.001), and autoimmune diseases (*p* < 0.001). At T2, a significant decreasing in anti-S IgG values was observed (187 [81–262] AU/mL), with a median decrease of 72 [60–82]%. On multivariate data analysis, a reduction of more than 82% was directly associated with male sex (*p* < 0.021), age (*p* < 0.001), smoking (*p* = 0.038), hypertension (*p* = 0.042), and, inversely, with previous COVID-19 infection (*p* < 0.001) and being “cohabiting” (*p* = 0.005). Our findings suggest that demographic, clinical, and social variables play a role in anti-S IgG values decreasing in long-term follow up and should be considered to find personalized vaccine schedules.

## 1. Introduction

The 2019 severe acute respiratory syndrome-coronavirus 2 (SARS-CoV-2) pandemic has led to an unprecedented rapid development of the mRNA-based BNT162b2/Pfizer- BioNTech vaccine against the SARS-CoV-2 virus. The BNT162b2 vaccine, a nucleoside-modified mRNA that encodes the SARS-CoV-2 Spike glycoprotein, elicits an immune reaction leading to the formation of long-lasting IgG immunoglobulins against the viral transmembrane Spike protein [1,2].

Several studies are currently focused on the long-term follow-up the SARS-CoV-2-specific humoral immune response. Immunological memory is the fundamental characteristic of the human adaptive immune system and the principle on which vaccination is based. Antibody decay is a highly heterogeneous physiological process among individuals [3]. In natural COVID-19 infection, antibodies can be detected from the second week but also as early as 4 days after the onset of symptoms. Anti-SARS-CoV-2 IgM and IgG seroconversion was observed around week 3 and week 4. IgM persists up to 5 weeks, declines, and vanishes almost completely by week seven [4]. IgG levels continue to be present up to eleven months after recovery [5].

Available studies suggest that the BNT162b2/Pfizer- BioNTech vaccine evokes an antibody-persistence up to 6 months with a peak at 28 days after the administration of the first dose followed by a progressive decline [6]. For this reason, monitoring the immune response against SARS-CoV-2 through the evaluation of IgG antibodies represents a tool to estimate the long-term vaccine efficacy [7,8].

Although the results of clinical studies suggest a similar vaccine response between individual subgroups [1], longitudinal studies have shown a different antibody decrease in real life studies [9,10]. Indeed, several factors have been identified to impact the decay of anti- SARS-CoV-2 IgG antibodies, such as older age, male sex, immunosuppression, high BMI, and smoking [10].

Here, we report the results of a real-life longitudinal study involving healthcare workers monitored for IgG antibody response after mRNA-based vaccination against SARS-CoV-2 (BNT162b2/Pfizer- BioNTech) from 1 to 5 months after a second dose vaccine. Thus, the main purpose of the study was to evaluate Anti-Spike (S) IgG levels and factors potentially influencing the long-term immunological reaction. This analysis could contribute to assess the duration and efficacy of the vaccine in specific categories of the population to provide an optimal protection against the infection and eventually to establish the timing for booster doses.

## 2. Materials and Methods

### 2.1. Participants

Health services workers with different commitments at Policlinico Umberto I—Sapienza, University of Rome, were enrolled in a longitudinal cohort study aimed at evaluatingn the temporal trend of serum antibodies against SARS-CoV-2 Spike. The study began in February 2021. Participants consisted of a heterogeneous population that included MDs, nurses, paramedics, administrative staff, and senior students attending the wards, who got the first COVID-19 vaccine between January and March 2021. All participants provided an electronic, signed informed consent form prior to participation in the study. Subjects self-administered an anamnestic questionnaire and provided blood samples for serological analyses; all data were de-identified. This research was reviewed and approved by the ethical committee of Azienda Ospedaliera Universitaria Policlinico Umberto 1 (ClinicalTrials.gov Identifier: NCT04844632).

### 2.2. Vaccination and Blood Specimen Collection

Before enrollment, all participants had received two COVID-19 vaccine BNT162b2 (Pfizer-BioNTech) inoculations, separated by 21 days.

All participants had blood drawn 1 month after the second vaccine dose. A sub-group of subjects provided blood samples for serological analyses also 5 months after the second vaccine dose.

### 2.3. Antibody Measurement and Interpretation

Antibody testing for SARS-CoV-2 Spike glycoprotein in serum samples was performed using an indirect chemiluminescence immunoassay (CLIA) that detects IgG to the trimeric form of Spike glycoprotein (SARS-CoV-2 Trimeric S IgG-LIAISON^®^- DiaSorin Inc., Stillwater, MN, USA) according to the manufacturer’s assay specifications.

The test provides quantitative results and permits to measure values between 1.85 and 800 Arbitrary Units per milliliter (AU/mL), corresponding to values between 4.8 and 2080 Binding Antibody Units (BAU/mL) (1 AU/mL = 2.6 BAU/mL). Positive anti-Spike IgG levels (TrimericS IgG) were defined as equal to or more than 13 AU/mL.

As reported by the manufacturer, the positive percent agreement of the LIAISON^®^ SARS-CoV-2 Trimeric S IgG test compared to PCR for samples ≥15 days from diagnosis is 98.7% (95% Confidence Intervals [CI] 94.5–99.6%), while the negative percent agreement is 99.5% (95% CI 99.0–99.7%) and the observed sensibility is >99%.

Moreover, the manufacturer provided guidance of SARS-CoV-2 anti-Spike IgG TrimericS reports the presence of neutralizing antibodies, confirmed by a 97.9% correlation with the microneutralization assay [11].

Antibodies against the SARS-CoV2 nuclear (N) protein were measured through a commercial sandwich electrochemiluminescence assay (ECLIA) (Elecsys^®^anti-SARS-CoV-2 assay, Roche Diagnostics, Switzerland). The assay targeting immunoglobulin (IgM, IgG, and IgA) provides a qualitative result with a sensitivity of 100.0% (95% CI 88.10–100.0) and a specificity of 99.81% (95% CI 99.65–99.91%). A cut-off index (COI) 1 is regarded as positive. As reported by the manufacturer, the assay has no biotin interference in serum concentration up to 1200 ng/mL

### 2.4. Statistical Analysis

Characteristics of participants were compared by sex, demographic and social characteristics, job duties in the field of health care, concomitant diseases, as well as by previous SARS-CoV-2 infection status. Categorical variables are reported as counts and percentages and continuous variables as mean ± SD, or medians and interquartile range (IQR). Differences between percentages were assessed by chi-square or Fisher exact tests. Continuous variables were compared using Student’s t-tests for normally distributed continuous variables. Appropriate nonparametric tests (Mann−Whitney U test, Wilcoxon signed-rank test Kruskal−Wallis H test) were used for all other variables. Categorical variables were compared by Chi-square test.

The bivariate and multivariate effects of prognostic factors on high levels of anti-S IgG and highest quartile of 5-months anti-Spike IgG reduction were assessed by means of logistic regression models. Wald confidence intervals and tests for odds ratios and adjusted odds ratios were computed based on the estimated standard errors. ANOVA models were used to analyze sex-interaction with demographic and social characteristics potentially influencing anti-S IgG at 5 months, using log-transformed anti-S levels as the dependent variable. All statistical analyses were performed utilizing using computer software packages (IBM SPSS ver. 27.0), and *p*-values <0.05 were considered significant.

## 3. Results

We recruited 2065 volunteers (age: 45.9 ± 13.3 years), consisting of 1307 (63%) women (age 44.7 ± 12.7 years) and 758 men (age: 48 ± 14.2 years), who were tested for the presence of SARS-CoV-2 Spike protein specific IgG antibodies after 1 (T1) and 5 months (T2) the second dose of the BNT162b2/Pfizer- BioNTech vaccination. The studied characteristics of the population are represented in Table 1. Most of the participants had a university degree. The occupational distribution was 50% medical doctors, 32% nurses, 12% general staff, 6% paramedics, and less than 1% students. In 26%, a smoking habit was present. The most observed clinical conditions were hypertension (18%), dyslipidemia (9%), history of asthma (6%), and autoimmune diseases (5%). One-hundred twenty-eight (6%) of the participants showed a previous COVID-19 infection (68 participants reported a previous infection in the anamnestic questionnaire, that, in 57 of them, was confirmed by anti-N IgG positivity; 56 participants demonstrated anti-N IgG positivity without reporting previous COVID-19 infection).

### 3.1. Anti-S IgG at T1 (1 Month after Second Injection)

**Figure 1:**
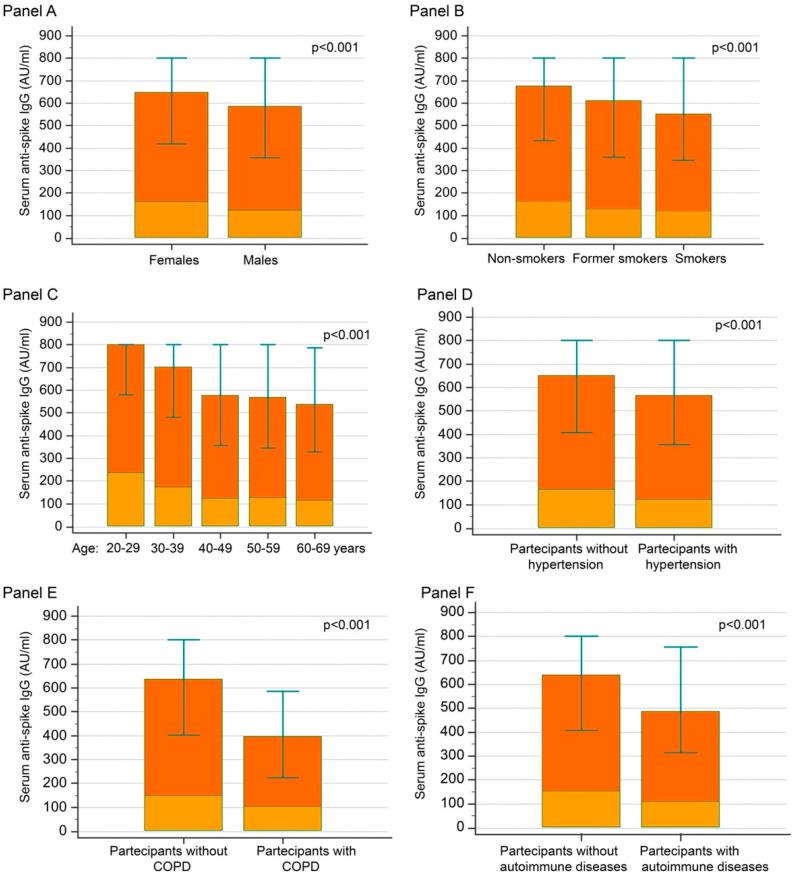
Anti-Spike IgG concentrations according to sex (Panel A), smoking habit (Panel B), age decades (Panel C), hypertension (Panel D), COPD (Panel E), autoimmune diseases (Panel F) at T1 and T2. Legend: Red boxes: median and IQR concentrations at T1. *p*-values: statistical differences at T1. Orange boxes: median concentrations at T2. 1 AU = 2.6 BAU.

At T1, the observed median and interquartile range (IQR) of the anti-S IgG values were: 693 (394; >800) AU/mL. Female showed higher values of anti-S IgG than males (Figure 1, Panel A). Similarly, non-smokers showed higher levels than smokers or former smokers (Figure 1, Panel B). A progressive decrease in anti-S IgG concentrations with increasing age decades was seen (20–29; 30–39; 40–49; 50–59; 60–69 years), although there were fewer differences in the subjects included in the age groups over 40–49 years (Figure 1, Panel C).

Furthermore, lower anti-S IgG concentrations were detected in subjects with hypertension, chronic obstructive pulmonary disease (COPD), or autoimmune diseases (Figure 1, Panels D–F).

**Figure 2:**
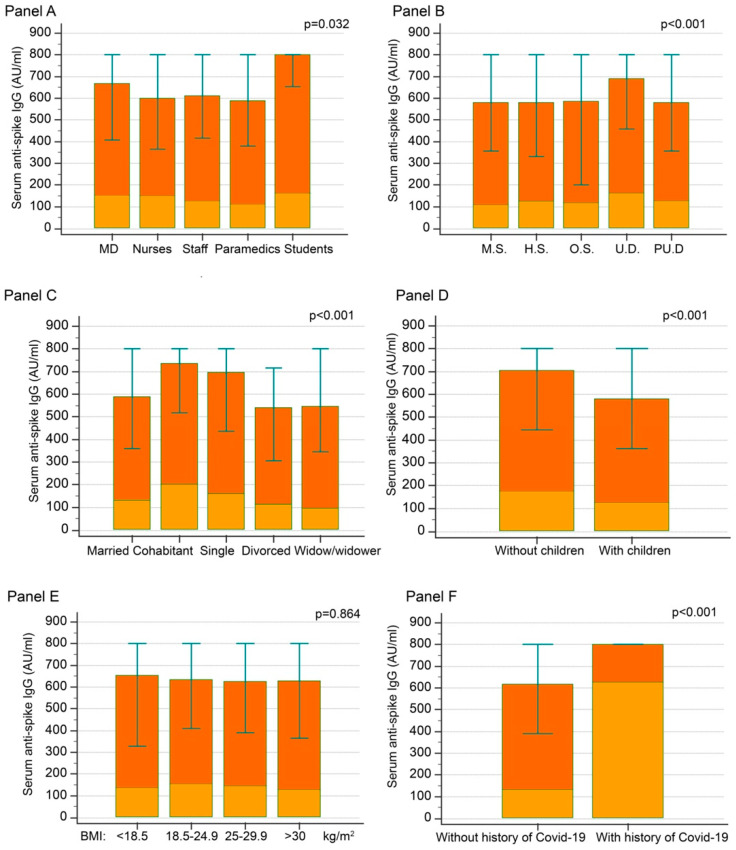
Anti-Spike IgG concentrations according to kind of occupation (Panel A), scholarship (Panel B), marital status (Panel C), childcare (Panel D), BMI (Panel E) and previous COVID-19 infection (Panel F) at T1 and at T2. Legend: Red boxes: median and IQR concentrations at T1. *p*-values: statistical differences at T1. Orange boxes: median concentrations at T2. 1 AU = 2.6 BAU.

Antibody distribution also differed between working roles, educational levels, marital status, and childcare (Figure 2, Panels A–D); medical doctors showed slightly higher antibody levels that other workers, such as the participants with a non-MD degree or without a degree. Interestingly, students demonstrated the highest humoral immunity versus MD and non-MD workers (*p* < 0.05). Regarding marital status, singles and cohabitants showed the highest values; participants with children showed lower levels than those without. No significant differences in anti-S IgG distribution were found in the different classes of BMI (i.e., <18.5; 18.5–24.9; 25–29.9; >30) (Figure 2, Panel E). Finally, participants with a history of COVID-19 infection (detected by the positivity of anti-N antibodies and/or self-reported) had higher levels of anti-S IgG with respect participants without a history of COVID-19 infection (Figure 2, Panel F).

Since 33% of the studied population showed anti-S IgG above the upper limit of the measurable value (800 AU), we divided the participants into two groups: those with anti-S IgG concentration above and those below 800 AU/mL (Table 1).

Participants with values more than 800 AU/mL were younger, more likely to be female, and to have a history of COVID-19 infection (anti-N IgG positive). Conversely, people with concentrations of anti-S IgG lower than 800 AU/mL were more likely to be smokers and to have hypertension, COPD, and autoimmune diseases.

Marital status and educational level of the volunteers were different among the two groups. Thus, people with serum concentrations of anti-S IgG above 800 AU/mL were more likely to be single or cohabitant and to have a university degree compared to those with values below 800 AU.

A multivariable logistic regression analysis showed that age, smoking habit, and autoimmune disease were inversely associated with values of anti-S IgG >800 AU/mL, while a history of COVID-19 infection was directly associated with the highest anti-S IgG values (Table 2).

Only three participants (0.14%) had concentrations of anti-S IgG less than 13 AU (two of them affected by an autoimmune disease).

### 3.2. Anti-S IgG at T2 (5 Months after Second Injection)

At T2, anti-S IgG was reevaluated in 1457 persons, giving median and IQR values of 187 (81; 262) AU/mL.

At T2, anti-S IgG was higher in female than in males (161 [92–290] vs. 125 [68–214] AU/mL; *p* < 0.001) and showed a progressive decrease with increasing age decades (20–30 years: 240 [147–393] AU/mL; 30–39 years: 174 [98–320] AU/mL; 40–49 years: 125 [75–214] AU/mL; 50–59 years: 128 [67–231] AU/mL; 60–70 years: 122 [68–209] AU/mL; *p* < 0.001).

Moreover, at T2, anti-S IgG was lower in people with hypertension (118 [68–200] vs. 159 [88–273] AU/mL in people without hypertension; *p* < 0.001), diabetes (102 [47–263] vs. 151 [84–260] AU/mL in people without diabetes; *p* = 0.023), ischemic heart disease (106 [35–177] vs. 151 [82–264] in people without; *p* = 0.034), autoimmune disease (110 [62–177] vs. 152 [84–266] AU/mL in people without; *p* = 0.009), and smokers (123 [65–201] vs. 130 [74–247] in former smokers and 163 [94–289] AU/mL in non-smokers; *p* < 0.001) and higher in people with a history of COVID-19 infection 624 [329–>800] vs. 139 [78–245] AU/mL in people without a history of COVID-19 infection; *p* < 0.001) (Figure 1 and 2).

Finally, anti-S concentrations at T2 showed significant differences according to marital status (single: 169 [97–308]; married: 130 [74–230]; cohabitant: 200 [129–337]; divorced: 110 [61–188]; widow/widower: 98 [54–242] AU/mL; *p* < 0.001), childcare (128 [73–231] vs. 174 [96–320] AU/mL in participants with or without children, respectively; *p* < 0.001), scholarship (university degree: 167 [98–292]; post-university degree: 132 [69–243]; high-school: 128 [64–242]; middle-school: 110 [58–204]; others 121 [60–224] AU/mL; *p* < 0.001), and according to the kind of occupation, with MD having significantly higher values than administrative staff (156 [86–272] vs. 131 [76–233] AU/mL; *p* = 0.033) and paramedics (111 [61–230] AU/mL; *p* = 0.034), while no significant differences were found with students or nurses (not shown) (Figure 2).

**Figure 3:**
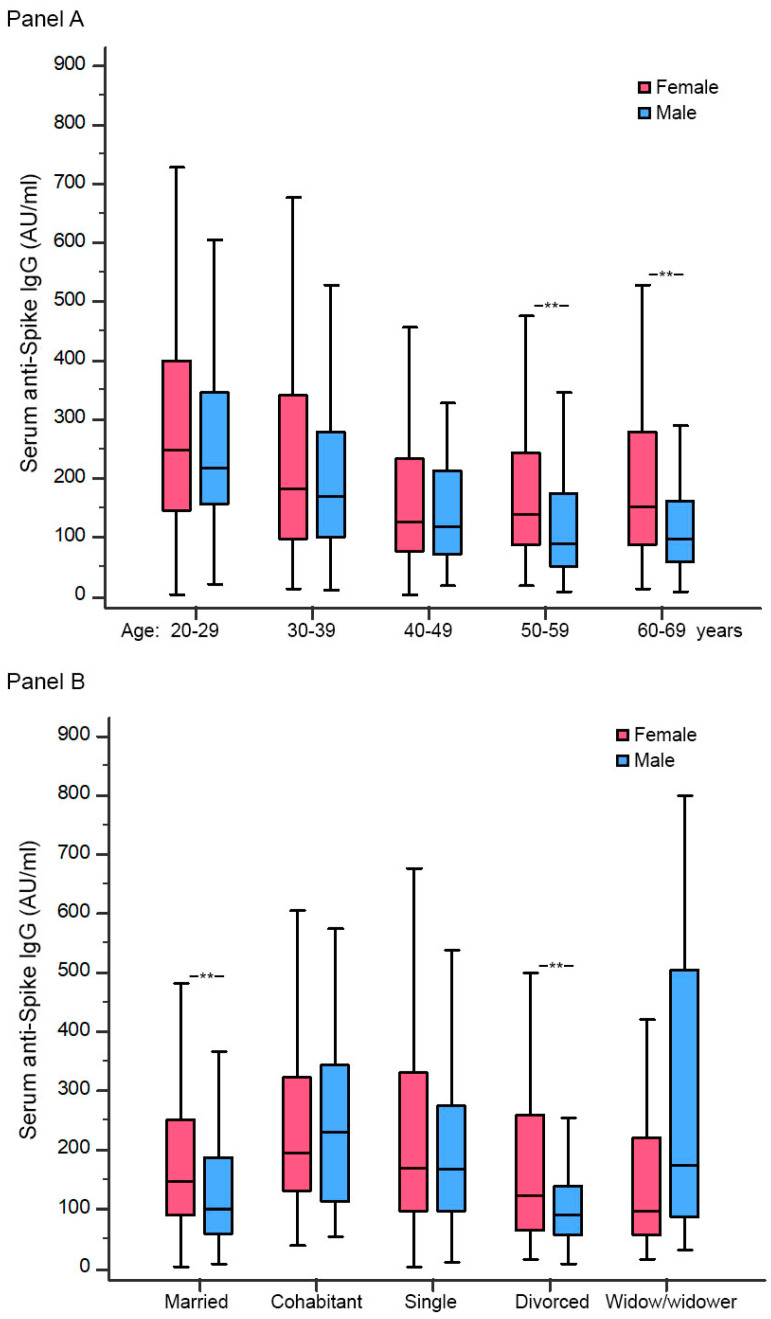
Anti-S IgG at T2 among male and female participants, according to age (Panel A) and marital status (Panel B). ** *p* < 0.001. Legend: 1 AU/mL = 2.6 BAU/mL.

ANOVA analyses showed significant effects of sex on IgG anti-S differences associated with age decades (F = 22.6; *p* < 0.001) and marital status (F = 2.6; *p* = 0.033). While females showed a progressive decrease from 20–29 years to 40–49 years without significant differences in the last decades, males showed a progressive decrease that continued in the elderly, showing significant lower values compared to females over 50 years old (Figure 3, Panel A). Regarding the marital status, married and divorced males showed significant lower anti-S IgG than females, while no significant sex-differences were found among singles, cohabitants, and widow/widowers (Figure 3, Panel B).

No significant effect of sex was found on IgG anti-S differences associated with childcare (*p* = 0.085), scholarship (*p* = 0.615), and type of occupation (*p* = 0.481).

**Figure 4:**
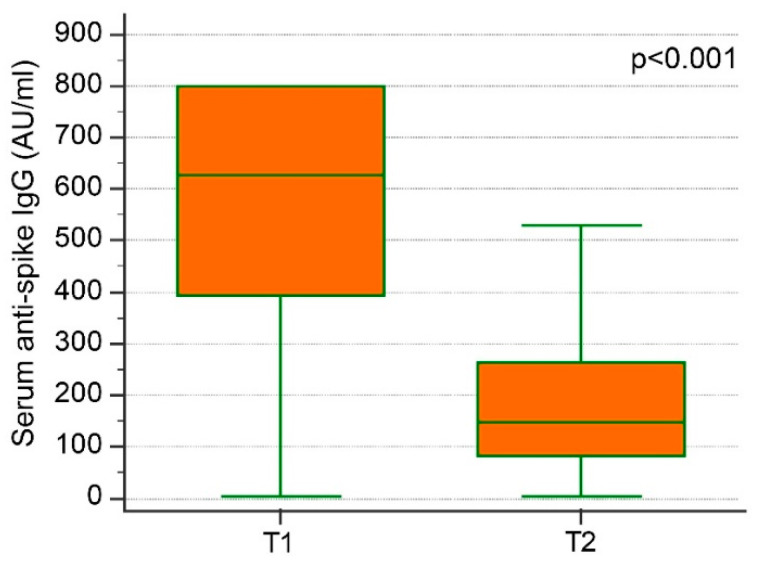
Anti-Spike IgG concentrations at T1 and T2 (i.e., after 1 and 5 months from second dose vaccine, respectively). Legend: 1 AU/mL = 2.6 BAU/mL.

From T1 to T2 the median anti-S IgG decrease was 72% (IQR: 60–82) (Figure 4).

A multivariable logistic regression analysis showed that a reduction higher than 82% (the highest quartile) was directly associated with age, male sex, smoking and hypertension, and, inversely, to a history of COVID-19 infection and to “cohabitant” marital status (Table 3).

Of note, 5 months after the second dose vaccination, only 10 participants (0.68%) showed anti-S IgG concentrations below 13 AU/mL.

## 4. Discussion

In this prospective longitudinal study, we reported a high immunogenicity of the vaccination, detecting a significant antibody production in more than 99% of cases both at 1 and 5 months after the second dose vaccine. At the same time, as expected [6,9,12], a significant waning of humoral response during the follow-up was observed, with a median reduction in anti-S IgG concentrations of almost four times in 4 months.

The study involved a relatively large cohort of healthcare workers (medical doctors, staff, paramedics, nurses, and MD-students) enrolled at the Sapienza University-Hospital Policlinico Umberto I, with the aim to investigate clinical, social, and demographic characteristics potentially influencing the short- and long-term immunological reaction to the vaccine.

At T1 (i.e., after 1 month from the second dose vaccine), the antibody answer was high—above the upper limit of the measurable value in about 33% of the participants. Participants with the highest antibody concentrations were younger, more likely to be female, and had a history of COVID-19 infection; conversely, smoking and the presence of comorbidities (i.e., hypertension, COPD, and autoimmune diseases) were associated with lower concentrations. Among the social characteristics, people with the highest concentrations were more likely to be single or cohabitant, childless, and graduated.

In a multivariate logistic regression analysis, the highest values of anti-S IgG at T1 remained directly associated with a previous COVID-19 infection and inversely associated with age, smoking habit, and autoimmune diseases.

At T2 (i.e., after 5 months from the second dose vaccine), a strong reduction in anti-S IgG was observed, with a median decrease of 72% and an interquartile range from 60 to 82%.

A reduction greater than 82% was independently associated directly with male sex, increasing age, smoking habit, and hypertension, and, inversely, with a history of COVID infection and being “cohabitant”.

The main clinical parameter that seemed to influence the antibody concentrations in our cohort was documented previous infection by SARS-CoV-2. People infected by the virus (as indicated by anti-N IgG positivity or anamnestic questionnaire) showed higher levels of anti-S IgG compared to infection-naïve individuals and were about eight times more likely to have anti-S IgG concentrations above the upper limit of the measurable value at T1.

These data are consistent with previous data from other cohorts indicating a strong association between antibody concentrations at short-term after vaccination and a previous SARS-CoV-2 infection [9,13,14,15].

However, the persistence of high antibody concentration in this setting is still a matter of debate: previous studies have showed both a more accentuated decline of antibody concentrations [13] and conversely a lack of drop in antibody concentrations at 3–6 months after vaccination if people were seropositive before vaccination [9,16]. Our study agrees with the latter, showing elevated anti-S IgG at T2 in participants with a previous infection. Moreover, these participants were five times less likely to be in the highest quartile of reduction during the follow-up, thus indicating that a previous infection is both associated with an enhanced anybody production and with a longer persistence.

In the present study, patients with an autoimmune disease showed lower anti-S IgG both at 1 and at 5 months after the second dose vaccination. Participants with autoimmune diseases usually obtain immunosuppressive drugs. Thus, this finding can be considered consistent with other reports, showing significantly lower antibody response after vaccination among participants with immunosuppression [9,17,18] and underlining the potential negative effect of immunosuppressive drugs in this context [8,18].

Moreover, antibody levels were higher in women than in men and decreased with age at both T1 and T2; this observation is consistent with published data in other cohorts [9,12,13,15,19] and clinical trials [1]. In addition, our analysis shows that sex and age seem to also influence the velocity of the decay that was more significant in elderly males.

In accordance with other cohort studies [20], vaccine-induced antibody concentrations were lower and decreased faster among smokers compared with nonsmokers. Our study strengthens this association, showing a progressive negative effect from non-smokers to former smokers to active smokers, suggesting a potential negative influence of smoking habit on immunity that cannot entirely end with smoking cessation.

In our study, hypertension predicted a more rapid lowering of anti-S IgG during the follow-up. To the best to our knowledge, only few clinical studies have found a strong relationship between hypertension and humoral response to COVID-19 vaccines [10], despite that hypertension has been constantly found to be related to worse COVID-19 outcomes [21], suggesting that the same metabolic features linked to higher mortality upon infection may be involved in the development of a low immunological response to the vaccination [10].

The novelty of the study is that we analyzed for the first time the relationship between some gender-related variables (such as educational level, job, marital status, childcare) and immunological response to COVID-19 vaccination. Although most of them resulted in being associated with different levels of antibody concentrations at univariate analyses, this finding was not confirmed in the multivariable analyses, indicating that other factors (for example, age, sex, and comorbidities) could explain these associations.

The only gender-related variable that remained associated with a lower decline in the immune response was “to be cohabitant”. This relation is difficult to explain at the moment. It may be possible that some other hidden clinical variable, not explored in this study, could account for the relationship. For example, we did not analyze the amount of visceral obesity and its impact on vaccine efficacy. In this regard, a recent study pointed out that central obesity, measured by waist circumference, but not BMI, was related to a poor antibody production upon vaccine administration [10]. Accordingly, no relationship between BMI and antibody concentrations was found in our study. Furthermore, we did not explore social behaviors that could have had an impact on the number of contacts with the virus during the follow-up.

When our study started, only 68 participants had reported a previous infection in the anamnestic questionnaire, while an additional 56 participants (82% more) showed anti-N IgG positivity, indicating that a large percentage of infections were asymptomatic. Thus, we cannot exclude that those new asymptomatic infections during the 4-month follow-up period could have increased anti-S IgG concentrations in some subgroups of people. Independently from the possible reasons of the relationship found, our analysis encourages further research to evaluate the impact of gender-related variables, as well as lifestyles, on immune responses.

In this study, we explored possible interactions between sex, age, and social variables associated with the long-term vaccine response, showing a sex-specific variability associated with age and marital status. A progressive decrease in anti-S IgG concentrations with increasing age decades was seen both in male and female younger people; after 50 years old, only males showed this trend, with significant lower anti-S IgG than females. Regarding the marital status, married and divorced males, but not singles and cohabitants, showed significant lower antibodies than females. The results of these analyses should only be considered explorative because of the relatively small sample size (for example, the number of widow/widowers is too low for subgroup analyses). However, similar observations about sex-specific age response have been observed in other studies [9]; thus, our finding supports further research exploring sex and gender interaction with clinical response to vaccines.

Our analysis presents some limitations. Even if the study included a broad kind of care workers with different social backgrounds, the cohort is from a single center and therefore may not represent the general population. As with any observational study, we cannot exclude the possibility that some of the associations we report might be explained by unmeasured confounding variables.

Finally, antibody concentrations may not reflect cellular immune responses, which should be analyzed to fully evaluate the protection to COVID-19 [22]. However, anti-S IgG concentrations correlate with neutralizing antibodies concentrations and possibly with protective immunity [9,23].

On the other hand, our study features some relevant strengths. To the best of our knowledge, this is the first study that simultaneously evaluated clinical and social factors associated with the immunogenicity of a COVID-19 vaccine and the first that evaluated anti-N IgG positivity to evaluate a previous infection in this context.

## 5. Conclusions

In conclusion, our data provide important insights into the longitudinal dynamics of the immune response to BNT162b2 vaccination. People may respond in different ways to the same vaccination; in this context, social and clinical factors as well as antibody evaluation may play an important role in predicting protection persistence. As the pandemic evolves, strategies to prolong host immunity need to be evaluated in order to protect the population against COVID-19 infection and the new SARS-CoV-2 emerging variants.

## Figures and Tables

**Table 1:** Demographic social and clinical characteristics of the vaccinated population and anti-S IgG serum levels at T1 (i.e., 1 month after the second dose vaccine).

Characteristics		Anti-S IgG (≥800 AU)	Anti-S IgG (<800 AU)	*p*
Age (years)	45.9 ± 13.3	41.9 ± 13.6	47.9 ± 12.8	<0.001
Female sex (%)	63.3	66.4	61.7	0.037
BMI (kg/m^2^)	26.1 ± 10.2	26.6 ± 12.9	25.9 ± 8.3	0.258
Age classes:				
20–29 years (%)	18.5	29	13.4	
30–39 years (%)	19.4	22.8	17.8	
40–49 years (%)	15.5	12.6	17	<0.001
50–59 years (%)	27.1	21.6	29.8	
60–69 years (%)	19.4	14	21.9	
Medical doctors (%)	49.8	53.8	47.6	
Nurses (%)	32.4	30.1	33.6	
Administrative staff (%)	11.6	10.3	12.3	0.069
Paramedics (%)	5.7	4.9	5.1	
Students (%)	0.6	1.0	0.4	
University degree (%)	54.2	59.3	51.5	
Post-university degree (%)	25.5	22.9	26.9	
High school (%)	15.6	13.7	16.6	0.032
Middle school (%)	3.5	2.8	3.9	
Other (%)	1.2	1.3	1.2	
Single (%)	40.0	45.9	36.8	
Married (%)	41.7	35.6	45.0	
Cohabitant (%)	9.3	12.3	7.7	<0.001
Divorced (%)	7.4	4.9	8.8	
Widow/widower (%)	1.6	1.3	1.8	
Having children (%)	52	43	57	<0.001
Smokers (%)	25.8	19.8	29.0	
Former smokers (%)	8.9	8	9.4	<0.001
Non-smokers (%)	65.3	72.2	61.6	
Hypertension (%)	18.5	14.9	20.4	0.004
Dyslipidemia (%)	9.4	8.3	9.9	0.279
T2DM (%)	2.1	1.8	2.3	0.502
Coronary heart disease (%)	1.0	0.7	1.1	0.322
COPD (%)	0.8	0.2	1.1	0.029
Asthma (%)	5.7	5.2	6.0	0.527
History of VTE (%)	1.1	0.8	1.3	0.349
Heart failure (%)	0.2	0.3	0.1	0.249
Chronic hepatic disease (%)	0.2	0.2	0.2	0.954
Chronic kidney disease (%)	0.3	0.0	0.5	0.072
Neoplastic disease (%)	0.6	0.5	0.6	0.743
Atrial fibrillation (%)	0.7	0.2	1.1	0.039
Autoimmune diseases (%)	5.2	3.3	6.2	0.008
Hematologic diseases (%)	0.8	0.7	0.9	0.616
History of COVID-19 (%)	6.0	14.3	1.8	<0.001

Legend: BMI: body mass index; COPD: chronic obstructive pulmonary disease; T2DM: type 2 diabetes mellitus; VTE: venous thromboembolism.

**Table 2:** Characteristics of the vaccinated population associated to elevated anti-S IgG (>800 AU/mL) at T1.

	O.R.	95% CI		*p*
Age decades	0.804	0.764	0.845	<0.001
Smoking habit	0.581	0.452	0.748	<0.001
Autoimmune diseases	0.552	0.327	0.930	0.026
History of COVID-19 (anti-N IgG and/or self-reported)	7.771	4.672	12.92	<0.001

After adjusting for sex, marital status, scholarship, childcare, and comorbidities.

**Table 3:** Characteristics of the vaccinated population associated with the highest decrease (>82%, T2 vs. T1) in anti-S IgG serum concentrations.

	OR	95% CI		*p*
Male sex	1.384	1.052	1.820	0.021
History of COVID-19	0.218	0.078	0.610	<0.001
Marital status: cohabitant	0.449	0.246	0.822	0.005
Age decades	1.171	1.080	1.271	<0.001
Hypertension	1.405	1.015	1.946	0.042
Smoking habit	1.376	1.021	1.855	0.038

After adjusting for comorbidities and scholarship.

## Data Availability

Not applicable.

## Appendix A

***SAPIENZAVAX Collaborators:*** Marcello Accordino, Luciano Agati, Riccardo Allerhand, Marco Anile, Marcello Arca, Marco Assenza, Ersilia Barbato, Alfredo Berardelli, Giuliano Bertazzoni, Marco Biffoni, Massimo Biondi, Maurizio Bossù, Stefano Brauneis, Diana Gabriella Bruno, Maurizio Bufi, Raffaella Buzzetti, Mauro Cacciafesta, Chiara Califano, Laura Capoccia, Marco Casadio, Milvia Casato, Matteo Casinelli, Carlo Catalano, Maria Rosa Ciardi, Fabrizio Consorti, Giuseppe Cimino, Serelina Coluzzi, Fabrizio Conti, Luca Cordaro, Bernadette Corica, Giancarlo D’Ambrosio, Vito D’Andrea, Alberto Deales, Maria De Giusti, Marco De Vincentiis, Giuseppe De Vincentis, Maria Del Ben, Stefano Di Carlo, Vittorio Di Piero, Cosimo Durante, Evaristo Ettorre, Giovanni Fabbrini, Francesco Fattapposta, Francesco Fedele, Mauro Ferrara, Enrico Fiori, Matteo Franchitto, Giorgio Franco, Fabrizio Maria Frattaroli, Ricciarda Galandrini, Gioacchino Galardo, Magda Gharbiya, Laura Giacomelli, Daniele Gianfrilli, Felice Giangaspero, Giuseppe Giannini, Corrado Girmenia, Francesco Gossetti, Paolo Gozzo, Antonio Greco, Cesare Greco, Teresa Grieco, Deborah Grilli, Gino Iannucci, Giulio Illuminati, Francesca La Gualana, Filippo La Torre, Antonietta Lamazza, Andrea Lenzi, Claudio Letizia, Vincenzo Leuzzi, Licia Manzon, Paolo Marchetti, Raffaella Marchettini, Bruno Marino Taussig De Bodonia, Maurizio Martelli, Domenico Mascagni, Claudio Maria Mastroianni, Enrico Mauriello, Sandro Mazzaferro, Gianluca Mennini, Silvia Mezi, Ivano Mezzaroma, Alessandra Micozzi, Stefania Mieli, Fabio Midulla, Marzia Miglionico, Andrea Mingoli, Salvatore Minisola, Fabio Miraldi, Santo Morabito, Maria Mura, Maurizio Muscaritoli, Livia Ottolenghi, Elena Pacella, Massimiliano Pacilio, Alessandro Paganini, Paolo Palange, Giorgio Palazzini, Andrea Palladino, Piergaspare Palumbo, Marco Paoloni, Giovanni Pellacani, Pasquale Pignatelli, Antonio Pizzuti, Giuseppe Placanica, Giorgio Pompa, Francesco Pugliese, Antonella Puzzovio, Isabella Quinti, Fabrizio Recchia, Diego Ribuffo, Paolo Ricci, Michele Roazzi, Massimo Rossi, Franco Gennaro Maria Ruperto, Bruno Salvati, Valter Santilli, Antonio Santoro, Enrico Sbarigia, Alessandro Sindoni, Salvatore Sorrenti, Leopoldo Spadea, Alberto Spalice, Lucia Stefanini, Gloria Taliani, Guglielmo Tellan, Francesco Tartaglia, Gianluca Terrin, Laura Tibaldi, Silverio Tomao, Elisa Tomarelli, Vincenzo Tombolini, Danilo Toni, Donatella Valente, Lorenzo Vantaggio, Federico Venuta, Massimo Vergine, Anna Rita Vestri, Annalisa Villa, Ciro Villani, Paolo Villari, Sara Vinciullo.

***SAPIENZAVAX—STEERING COMMITTEE:*** Antonio Angeloni, Giulio Antonelli, Alberto Deales, Claudio Maria Mastroianni, Anna Rita Vestri, Paolo Villari, Laura Tibaldi.

***SAPIENZAVAX—DATA AND SAFETY MONITORING BOARD:*** Stefania Basili, Manuela Di Franco, Alessandra Zicari, Roberto Cangemi, Marcella Visentini, Vincenzo Cardinale, Giulio Francesco Romiti

***SAPIENZAVAX—SAMPLE COLLECTION, STORAGE, AND ANALYSIS CENTER:*** Antonio Angeloni, Giulio Antonelli.

***SAPIENZAVAX—PROMOTORS:***

Sapienza University of Rome (Rector: Antonella Polimeni);

Faculty of Pharmacy and Medicine, Sapienza University of Rome (Dean: Carlo Della Rocca);

Faculty of Medicine and Dentistry, Sapienza University of Rome (Dean: Domenico Alvaro);

AOU Umberto I, Rome (General Manager: Fabrizio d’Alba).
